# Technology and clinician-learner interaction: how clinicians expect introduction of a new electronic health record to affect educational practice

**DOI:** 10.1186/s12909-022-03925-3

**Published:** 2023-01-10

**Authors:** Julianna Caon, Kevin W Eva

**Affiliations:** 1 BC Cancer Victoria, Victoria, Canada; 2 Centre for Health Education Scholarship and Department of Medicine, University of British Columbia, Vancouver, Canada

**Keywords:** Electronic health records, Medical education, Clinical teaching

## Abstract

**Introduction:**

Electronic health records (EHRs) are increasingly common platforms used in medical settings to capture and store patient information, but their implementation can have unintended consequences. One particular risk is damaging clinician-learner-interactions, but very little has been published about how EHR implementation affects educational practice. Given the importance of stakeholder engagement in change management, this research sought to explore how EHR implementation is anticipated to affect clinician-learner interactions, educational priorities and outcomes.

**Methods:**

Semi-structured interviews were conducted with a group of practicing oncologists who work in outpatient clinics while also providing education to medical student and resident trainees. Data regarding perceived impact on the teaching dynamic between clinicians and learners were collected prior to implementation of an EHR and analyzed thematically.

**Results:**

Physician educators expected EHR implementation to negatively influence their engagement in teaching and the learning they themselves normally gain through teaching interactions. Additionally, EHR implementation was expected to influence learners by changing what is taught and the students’ role in clinical care and the educational dynamic. Potential benefits included harnessing learners’ technological aptitude, modeling adaptive behaviour, and creating new ways for students to be involved in patient care.

**Conclusion:**

Anticipating the concerns clinicians have about EHR implementation offers both potential to manage change to minimize disruptions caused by implementation and a foundation from which to assess actual educational impacts.

**Supplementary Information:**

The online version contains supplementary material available at 10.1186/s12909-022-03925-3.

## Introduction

Electronic health records (EHRs) are a technology-mediated way for health care professionals to safely store and access patient data [1]. Their use is increasingly common due to their clinical benefits such as enhanced ability to follow guideline-based care, facilitating more robust surveillance programs, and reducing medication errors [2, 3]. Despite these apparent advantages, a systematic review of the literature regarding EHR implementation has shown transition to their use to be anything but a benign event as some institutions struggle with EHRs changing the culture of the workplace and impairing communication [4].

While of central importance, direct clinical work is not the only activity in which physicians in academic settings are engaged; nor is it the only way in which clinicians impact upon the long-term healthcare provided to a community. Rather, physicians are expected to provide meaningful workplace-based learning experiences for trainees in support of the next generation of medical professionals. Given the imperfectly overlapping needs of patients, providers, and trainees, complex and challenging tensions can arise at the best of times when trying to support both learner education and patient care [5, 6].

According to Tierney et al., EHRs can increase a learner’s medical knowledge through exposure to clinical decision-making support systems [7]. Conversely, these same systems can interrupt workflow and may, thus, alter capacity for workplace-based learning [7]. The research conducted to date, however, has been largely silent on how EHRs influence clinician-learner interactions or how such effects might impact upon educational outcomes. This is an important gap to fill for anyone hoping to engage in EHR-related change management; the more we engage stakeholders to anticipate their concerns and where resistance may arise, the more we stand to optimize efforts to make change in a way that is both safe and educationally meaningful [8, 9].

In 2012, Spencer et al. offered some insight into the question of how EHRs affect education by reporting that implementation of a new EHR distracted physicians from teaching and reduced their enthusiasm for that activity [10]. While offering interesting leads, the authors’ use of a post-implementation survey with negative declarative statements carries the risks that negative ideas were planted in respondents’ minds and that the constraining nature of surveys prevented the researchers from seeing things they did not explicitly ask about.

In an effort to triangulate upon and broaden these findings, we adopted a more open-ended methodology with a group of participants in a different practice context who were asked to reflect on the impact EHRs can be expected to have. While exploring the issue at a pre-implementation stage should not be mistaken as providing an indication of actual post-implementation experiences, stakeholder expectations are important to study in their own right by virtue of providing valuable guidance regarding where intervention is likely to be required during change management [8, 9]. It also broadens the focus of exploration by avoiding reactions that are specific to the success or failure of a particular implementation effort. In doing so, such exploratory work can lay the groundwork for further study as the first stage of a design-based research project in that the findings can be used to allow better understanding of what to look for when subsequently evaluating clinician-learner interactions during and after EHR implementation [11].

Conceptually, this work aligns with sociomateriality theory [12], as will be explained below, in that it is the first stage of an exploration into how a piece of technology (i.e., a material) is liable to influence social behaviour (student-preceptor interactions) in the learning environment. Specifically, the aim of this particular study is to investigate how the introduction of electronic health record technology is expected to affect the teaching dynamic between clinicians and learners from the perspective of physicians who routinely engage in teaching medical trainees.

## Methods

### Design

We were guided by generic qualitative methods given the desire to remain flexible and broadly exploratory [13]. In doing so, we adopted a constructivist outlook because the fundamental goal of this study was to understand physicians’ beliefs about anticipated impacts of EHR implementation on clinical teaching and, hence, the data can be expected to be grounded in the physician educators’ perspective and co-constructed through discussion with (and analysis by) the investigators. JC is a practicing oncologist with the cancer centre from which the sample was drawn. She, thus, can be considered an insider who was herself required to anticipate how her teaching might need to change upon implementation of an EHR in the clinic. This offered deeper insight into the context in which the study took place. To help guard against her impressions becoming self-fulfilling prophecies, however, she worked closely with KE, a non-clinician education researcher, when designing the interview guide, reviewing the interviews, coding, and analyzing the data. Through repeated and iterative conversations, they challenged one another’s interpretations and achieved consensus regarding the core themes to be drawn from the study.

#### Setting

The population studied was the group of practicing radiation and medical oncologists working within BC Cancer, the provincial cancer centre for British Columbia, Canada. This was considered an ideal group to help address the issue at hand because there is an upcoming agency-wide roll-out of a new EHR. The recruited physicians work as clinicians in out-patient clinics that include postgraduate trainees and often provide workplace-based training rotations for medical trainees during their clinical clerkships. As a result, they are experienced clinical preceptors and, thus, key informants regarding the variables that influence their capacity to provide care and educate trainees in a clinical setting. Pre-EHR implementation, the outpatient clinic uses a hybrid approach with paper record keeping and a variety of electronic platforms being used by individual clinicians. Learner access to the electronic platforms is limited such that any teaching that draws upon clinical records is likely to take place using paper charts or clinical teachers’ own knowledge of pertinent patient information.

### Sampling

Recruitment entailed sending an introductory email to all 34 practicing radiation (*n* = 17) and medical (*n* = 17) oncologists working at the Victoria site of BC Cancer. We sought maximum variation sampling within this context in an effort to reflect the breadth of understanding and perspective on the topic [14]. Therefore, based on the initial response to the invitation email, follow up contact was deliberately directed at recruiting a range of individuals defined by various ages, years of practice, involvement with EHR development, and the tumor sites they treat/clinical teaching they perform. When no new themes could be extracted from the data, we ran one additional interview as confirmation that saturation had been achieved and then stopped sampling.

#### Data generation

All interviews were semi-structured and conducted one-on-one by the first author with notes taken at the time of interviewing. Interviews were conducted over a 5-month time period and were voice recorded and transcribed.

The interview guide was created through repeated discussion between investigators and continually modified after the early interviews to improve clarity and focus and to reduce the risk that participants were led towards particular responses (see Additional file 1 Appendix 1). All interviews began by exploring the background knowledge of the participant regarding the upcoming EHR implementation and their current involvement with clinical education. Later questions probed what participants anticipated would happen to their teaching dynamics during the EHR implementation process, what adjustments those changes would require, and what resources/barriers would likely influence clinical educators’ capacity to make any necessary adjustments.

### Data analysis

The voice-recorded interviews were transcribed by an external third party and anonymized prior to analysis. The first three transcripts were read and coded by both authors with comparisons made to discuss differences of approach, focus, prioritization, and interpretation. All transcripts were subsequently read and fully coded by the first author with routine and iterative checks made by the senior author and an effort made to engage in constant comparison (i.e., routine testing of new ideas against pre-existing findings and previously analyzed data).

This process began by reading the transcripts and assigning tentative labels to statements of interest as per the notion of ‘open coding’ [15]. We then engaged in axial coding by comparing the categories generated to one another to identify redundancies, similarities, and differences in an effort to extract a refined set of core themes. Finally, selective coding was employed by re-reading the transcripts to deliberately identify all data that related to the core themes while allowing further and deeper analysis of the inter-relationships between the categories identified [15]. During this stage, we were able to verify our understanding of the data by trying to apply the themes that had been created to the raw data while deliberately seeking counter-examples.

Throughout the coding process, we attempted to conceptualize the data by constantly asking the question “What is happening here?” as per the advice of Watling & Lingard [14]. As such, analysis continued during the write up of the results as the effort required to describe and cohesively present the data allowed new understanding of their interrelationships and organization.

## Results

Eight practicing physicians participated in this study, 5 radiation oncologists and 3 medical oncologists. Seven of eight identified as female and experience ranged from 2 to > 15 years in independent clinical practice. All actively taught learners in the current clinical environment at least once a month and were aware of plans for a new EHR to be implemented. The level of detail regarding their understanding varied from having no knowledge of how the EHR implementation would be conducted to being involved in the groundwork for its creation. Participants generally expressed drawing enjoyment from teaching medical trainees in clinic.

After data analysis, two core themes were developed. We found that EHR implementation is anticipated to have pervasive effects on the teaching dynamic in outpatient clinical settings that was best conceptualized in terms of how the teacher is impacted and how the learner is impacted.

### Theme 1: impact on the teacher

**Fig. 1:**
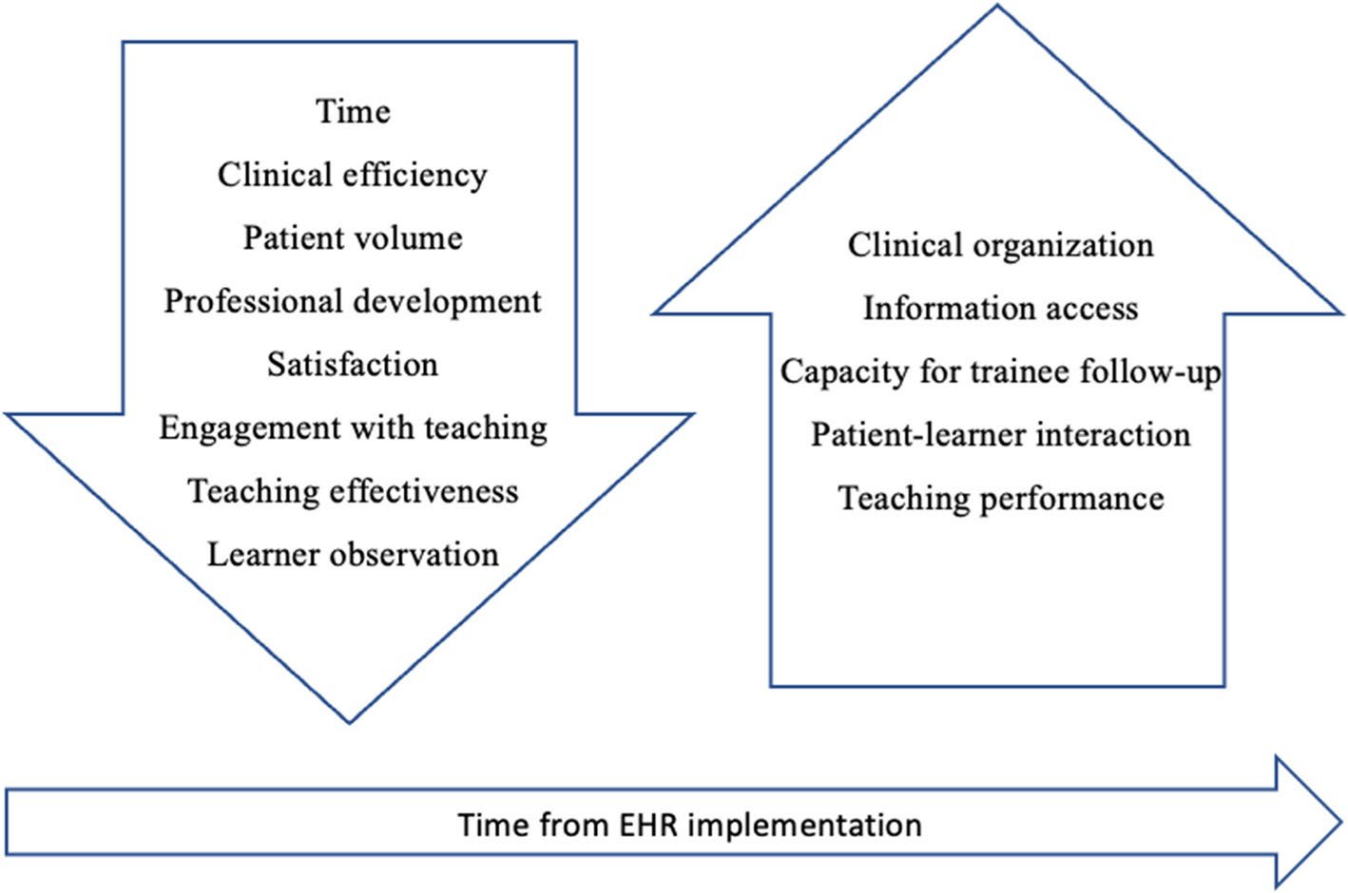
The anticipated impact of EHR implementation on teacher

The impacts of EHR implementation that participants in our study anticipated experiencing fell into two categories that are illustrated in Fig. 1: professional development (i.e., reductions in personal learning and capacity to teach) and engagement (i.e., feeling de-motivated to teach, balanced against better access to clinical information that might improve one’s ability to teach).

#### Professional development

Participants expressed a belief that the already scarce resource of time will be stretched even more thin by trying to navigate the EHR system during its implementation. Physicians expected to feel rushed and, therefore, did not anticipate having the time they would normally have to spend with learners in clinic. This change was not only expected to reduce their capacity to teach how and what they wanted to learners, but was also thought likely to affect the personal learning they normally gain during teaching interactions.

That is, participants worried that the gains they achieve by having learners, who routinely encourage them to “go back to basics,“ in clinic would be lost, thus impacting on their practice of continuing professional development and lifelong learning.

I think [having less interactions with a learner during EHR implementation] takes away from the learning experience for both of us. (Participant 3)

In having such an effect, EHR implementation was anticipated to create additional pressures that run counter to recently adopted competency-based medical school and residency curricula, which emphasise observation of learners’ clinical interactions. As a result, teachers worried about the extent to which they would be able to do their job as educators effectively.

…the curriculum has changed that way, in that there’s desire for clinicians to directly assess learners, but I think that it will be really difficult to get people to do that in the implementation for certainly the first three months. (Participant 4)

#### Engagement

Throughout the interviews, this concern about teaching well led physicians to share a number of ways in which they thought EHR implementation would mute their capacity to engage with students, including anticipation of several system-wide changes such as the need to adjust bookings to allow more time between patient encounters when learners are in clinic. As a means to overcome such concerns, participants suggested that trainees be included in the process of building the EHR and recommended that they be provided adequate training on the EHR before their time in clinic. Such suggestions, however, were offered with an underlying tone that such strategies were unlikely to be undertaken in reality. That is, it was expected that physicians would just need to absorb the extra work and pressures associated with EHR implementation, shouldering the responsibility to “do it all” and negatively affecting their engagement with teaching.

One thing that I’ve noticed observationally in myself and my colleagues is that we try not to do less. And so, we would continue to try to do as good a job clinically and as good a job educationally, despite the hurdles that are placed in our way. And so, I think it will simply become more difficult to teach in that way … I think I would have less satisfaction in teaching because it comes at a greater cost. (Participant 1)

These findings yield hints, however, regarding how engagement in teaching might be maintained given that participants were able to identify some positive anticipated impacts of EHR implementation. Some saw the potential to improve their teaching by virtue of the EHR better organizing clinical information, thereby allowing them to follow up on the clinical work of the trainees better. Others felt that having computers in patient exam rooms would change their ease of access to information, thus enabling better teaching through direct engagement with learners while the patient is present. These optimistic outlooks were accompanied by hope that the new EHR would eventually improve clinician efficiency, resulting (again, eventually) in more time to provide the clinical teaching they desire giving.

I think the goal is that it’ll be more efficient, so if it does eventually become more efficient, well then it might be that…there is eventually going to be more time for teaching. (Participant 2)

#### Theme 2: impact on the learner

**Fig. 2:**
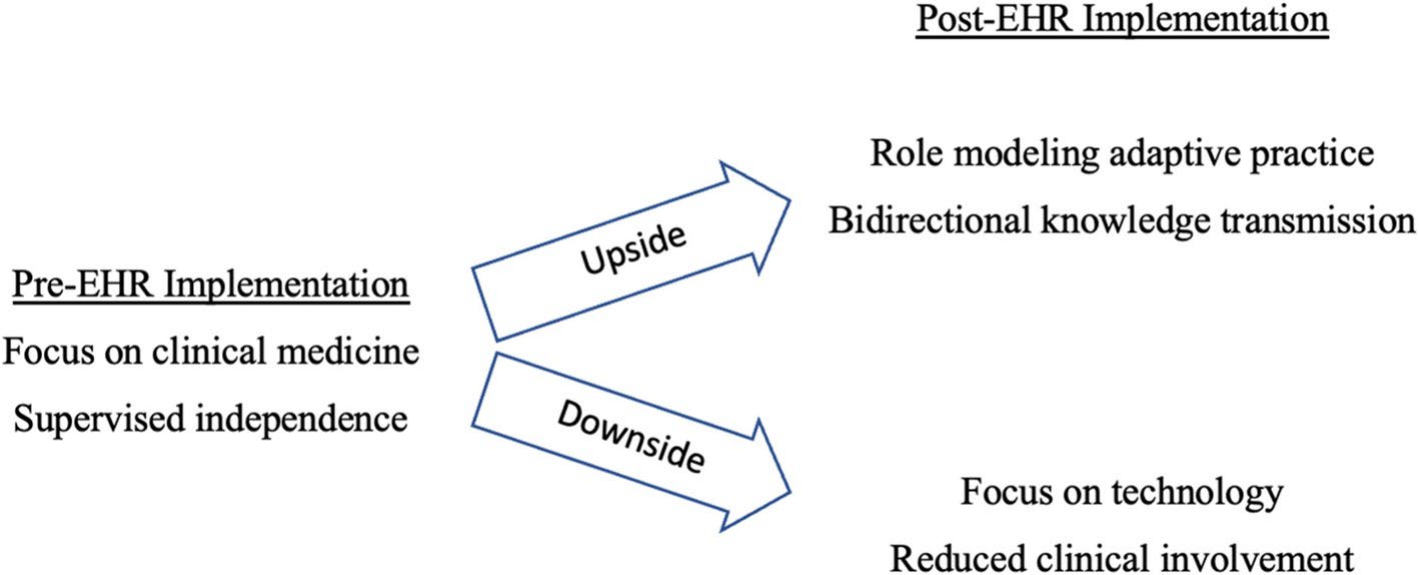
The anticipated impact of EHR implementation on learners

Figure 2 illustrates the positive and negative affects of EHR implementation on clinician-trainee interactions anticipated by our physician participants. They noted that a large component of clinical teaching involves the learner having some degree of involvement and supervised independence in clinic, particularly in the ability to write orders and dictate notes. It was acknowledged that learners are generally more technologically savvy than their faculty, but physicians were uncertain regarding what access or training learners would receive within the new EHR system. There was strong concern expressed that if learners did not have adequate access to the system, they would be less clinically involved, thereby negatively impacting their learning.

It was argued that a lot of teaching about clinical medicine currently involves the physician modelling clinical practice and decision making, which might be disrupted by greater focus on the incorporation of technology, or could create bad habits on the part of new clinicians.

As we become computer centric, mechanistically-driven professionals, as opposed to humans interacting with humans that are actually extracting a story… as opposed to pursuing data, that will be modelled earlier and earlier at school curriculum. (Participant 1)

What learners would be learning, therefore, could fundamentally shift as having a new EHR would distract from teaching about clinical medicine, creating more emphasis on learning about a specific technology.

[EHR implementation will] take a little bit away maybe from focusing on like the content of oncology, and a little bit to like some other – a little bit maybe more of a focus on logistics. (Participant 2)

The potentially positive side of such a shift in focus, expressed by some physicians, is that during EHR implementation physicians may have more opportunities to role model other types of competencies expected of care providers, including how to manage a stressful environment, resiliency, and lifelong learning.

I think that there is a lot to observe in terms of, you know, … resiliency, and… yeah, some burnout prevention and things, too, now, because the same things are happening. Like there’s always external stresses, there’s always change…So I think we have to model, you know, how we’re managing that, and I think it’s important for them to see that that stuff like that. (Participant 6)

Given the possibility that what learners can be expected to learn would shift during a major change in the clinical workplace, it was also expected that learners could take on a more central (albeit different) role in clinical care and learner-teacher interactions. The potential to impart their comfort with technology to the clinician, that is, could similarly allow for a more bi-directional transmission of knowledge and skill that could, in turn, allow the learner to feel more integral to the clinic.

I’m pretty open about the stuff I don’t know, and I prefer to learn how to do it, rather than pretend to know how to do it. And I think the learner-teacher relationship is two ways. (Participant 7)

## Discussion

With this study, we sought to examine how EHR implementation is anticipated to affect clinician-learner interactions and, in turn, impact upon educational priorities and outcomes. In doing so, we found a series of interesting dichotomies suggesting that for every unintended consequence there is potential for an equal and opposite positive reaction, reminiscent of the adage that in every crisis there is opportunity. On one hand, clinical educators worry about losing the advantage of teaching as a means of updating their clinical knowledge; on the other, some saw the possibility of improving their care by drawing on the technological sophistication of their trainees. On one hand, clinical educators worry about heightened focus on technology harming the professional identify formation and patient-centred focus of their trainees; on the other, some saw the opportunity to model resilience and the need to constantly adapt one’s practice. On one hand, clinical educators worry that the shift to an EHR will push learners to the periphery of clinical experience; on the other, it was thought to create ways for them to more readily take on central responsibilities in patient care.

In a recent time motion study performed with emergency room physicians, overall time spent doing “direct teaching” and “technical/logistical teaching” did not change pre, during, and post EHR implementation, which is a promising result [16]. Our study, however, suggests that there is more than just the marker of time to consider as balancing the above listed dualities in the teaching dynamic are likely to need careful consideration and monitoring during EHR implementation.

At their core, these findings can be summarized as the belief that EHR implementation is expected to require educational adaptation because it is likely to alter teachers’ ability to prioritize teaching, to engage with learners in tried and tested ways, and to provide adequate coverage of the clinical skills and knowledge believed to be critical competencies derived from training in this context. Participants believed EHR implementation may change what is modelled by physicians, trainees’ involvement in the clinical workplace, and the reciprocal dynamic between trainees and physician educators. In the language of sociomateriality theory, technology and social interactions were anticipated to be integral to one another EHRs were thought likely to act as a “material” that has the capability to affect the learning environment quite unintentionally by setting priorities in ways that influence learning [12]. This observation reinforces recent calls to study status quo workflow [17] and stakeholder perceptions as an important part of understanding how technology deployment might change practice because whether the technology’s effects are positive or negative, on balance, is likely to depend on the extent to which proactive planning through careful change management can shift learners’ experiences from the latter to the former.

For example, the simple presence of an EHR, and the time and cognitive resources required to navigate it, is expected to be capable of reducing the teaching physicians consider to be important (reducing feedback, direct assessment, and one-on-one time). That would be particularly disconcerting in the context of recent efforts to make curricula more competency-based (requiring physicians to be interactive with learners). This leads to the inherent risk of lessening opportunities for trainees to benefit from the experiential learning that is crucial both to skill development and professional identity formation. Given that our group of participants described themselves as those who enjoy and get fulfillment from their clinical teaching obligations, careless EHR implementation runs the risk of de-motivating the very group of clinicians who are particularly desirable to educational programs and on whom educational programs are particularly dependent. It is, therefore, imperative that clinical and educational leaders take steps to encourage and facilitate the optimism expressed by those who hoped EHR implementation could improve engagement in teaching by harnessing the technology to organize information or facilitate teaching more directly than often occurs in haphazard clinical environments.

These results, combined with a variety of theoretical concepts from education more generally, provide a framework for guiding such efforts as well as for monitoring the breadth of educational impacts that might be felt upon implementing an EHR. Here we will outline two examples.

First, prior research suggests that the implementation phase of a new technology is a particularly challenging time during which unrecognized “materials” can lead to implementation failure [18]. In our study, the way in which records are kept, controls over who has access to such records, the need to learn how to interact with a new technology and changes to the physical orientation of the clinic were all described in ways that suggest they would “assemble” to enact change in both how teachers engage with learners and what educational outcomes are obtained. This emphasis on sociomateriality also reflects that evaluation and management of the changes experienced need to be monitored over a period of time rather than trusting that initial impacts will be felt forevermore.

Second, Lave and Wenger’s seminal work on communities of practice theory is likely to be useful to sensitize educators to the notion of enabling legitimate peripheral participation as a means to move trainees towards the centre of their chosen community through development of professional identity [19]. In our study, the EHR was thought to create risk to this transition, but participants also provided hints as to how trainees could be helped to gain different skills through engagement in tasks that directly contribute to care both by helping the physician to keep accurate patient records and by using their own technological know-how to help their clinical preceptors continue their own professional development. The observed variety of impacts emphasizes the need to monitor an array of potential outcomes rather than focusing purely on aspects of care like medical knowledge.

In more proactive practical terms, it is worth noting that Spencer et al. reported physicians being most affected by EHR implementation when they were particularly enthusiastic teachers pre-implementation and being least affected when they were particularly comfortable with the new EHR [10]. Combining their findings with ours suggests that, if we want to keep physicians fulfilled in their educator roles, ensuring adequate training on the EHR, as well as providing resources to help physicians cope during implementation, should be a priority. This goes for learners as well as their preceptors as our participants worried that if trainees do not have complete access to the new EHR, it would reduce their ability to be clinically independent and reduce their resultant opportunities to learn. Incorporating medical trainees into the EHR design team, as well as ensuring their adequate training and feedback could further minimize the potential for negative impacts of EHR implementation on educational outcomes [20].

In summary, direct implications that can be drawn from our findings include considering context, content, and processes when approaching the complex matter of change management in the context of EHR implementation. If feasible, reducing non-essential physician tasks and patient load during implementation may lead to more time for physicians to work with the new EHR, see their patients, and continue to provide teaching to learners in clinic. Incorporating the learner into the EHR implementation framework, to ensure they too have adequate access to the system, may improve their capacity to remain actively involved in clinic during and after implementation so as not to erode their learning experience.

That said, there are several limitations to this study that should be noted. First, it was conducted in a specific group of outpatient oncologists working in Canada that happened to be skewed towards female gender and, thus, the perceptions obtained may not be transferable to different groups. We attempted to maximize the heterogeneity of participants to reduce this risk, but the need to draw from a population that was anticipating EHR implementation in the near future limited the pool of participants available; why women were more likely than men to respond to the invitation (and what impact that might have had on the findings) is unclear. Second, as participants were asked to anticipate an event that has not yet happened, our data may reflect an over or underestimate (or mixture of both) of the impact EHR implementation has on teaching dynamics. They provide a strong foundation, however, from which determine factors that need to be considered during change management and for adequate evaluation of impact when implementation does occur.

## Conclusion

This study has shown that physician educators anticipate EHR implementation will influence teaching dynamics in a way that impacts upon both the teacher and the learner. In doing so, it provides guidance regarding the variety of features that need to be managed and monitored during change processes to enable the continuation of effective learning and to evaluate implementation effectiveness. Quantifying the time spent by the teacher interacting with learners before, during, and after implementation may be important, but it may also be advantageous to specifically observe the content being taught and the bidirectionality of teaching interactions. Watching for changes in interest or motivation to teach throughout implementation and for changes in the direct clinical involvement of trainees is likely to be important as our findings suggest that EHR implementation may not only influence what is formally taught, but is also likely to drive the hidden curriculum, particularly in regard to what skills are role modelled and what educational activity is valued over time.

## Supplementary Information

**Appendix 1.**

## Data Availability

The datasets generated and/or analysed during the current study are not publicly available in order to not identify participants but some data can be made available from the corresponding author upon receipt of a reasonable request.
