# Supplementary material for: Technology and clinician-learner interaction: how clinicians expect introduction of a new electronic health record to affect educational practice
Source: BMC Med Educ. 2023 Jan 10;23:14. doi: 10.1186/s12909-022-03925-3 (PMC9830692; doi:10.1186/s12909-022-03925-3)
Supplement: Supplementary file 1 — Appendix 1. [file 12909_2022_3925_MOESM1_ESM.docx]

**Appendix 1**

**Interview Guide**

1. The main thing I would like to talk with you about today is what you expect the implementation of a new electronic health record system to do with respect to your practice as a clinical educator.
   1. Do you expect the implementation of Cerner to influence your teaching in any way, for better or worse, in the short or the long-term? What **changes in teaching** do you anticipate?
      1. Probe for changes in quantity (ie. will the time spent teaching go up or down), activity (ie. will the type of teaching change), focus (ie. will the content or intention of teaching change).
      2. Probe for who is going to be influenced (clinician, learner, other?). Are the changes described, if any, more likely to impact upon postgraduate (resident) or medical student trainees?
      3. Probe for the participant’s reasoning – why is the EHR expected to have the influences named?
      4. Probe for how the geographic changes associated with EHR (ie. Computer access in exam rooms) may impact teaching dynamics
   2. What impact will the **changes in teaching** that you anticipate have on you, your students, or your patients?
      1. Probe for whether the changes named are expected to be long term issues or moments of adjustment
      2. Probe for how concerned the participant is regarding the potential impact (i.e., will the change be a minor annoyance or a fundamental shift that will require massive change)?
      3. Probe for what they think medical trainees may learn from clinicians during this implementation (ie. issues regarding modelling new skill development and lifelong learning)
   3. How might clinical teachers be supported through implementation of a new electronic health records system?
      1. Probe for issues regarding time constraints, feelings of embarrassment for ‘not knowing’, stress factors, the idea of ‘expertise reversal’
      2. Probe for what guards could be put in place to protect against any negative impacts for which concern is expressed. Why are the guards named expected to be effective? Are they thought to be high priority?
      3. Probe for how to maximize the benefits of any improvements anticipated
      4. Probe for how the new EHR might influence medical training at a systemic level, in the context of the overall education system within this institution rather than the just clinician’s direct student interactions (ie. Do they think there will be any need to adjust how medical trainees in clinic are scheduled, or engaged with, during or after implementation of the new electronic health records system?)
2. If we were to extend this study by trying to actually observe if or how teaching changes during the upcoming EHR implementation, what would you suggest we pay particular attention to?
   1. What would your hypothesis be regarding whether EHR implementation would be beneficial, detrimental, or not influential?

i. Probe what educational processes should be examined and what outcomes might be influenced given the rationale expressed for what influence EHR implementation is likely to have. Probe why those beliefs are expressed.

1. Are you planning to do anything different in your educational practices in an effort to guard against any detrimental effects that might occur during EHR implementation?
2. Keeping in mind that the goal of our conversation is to understand if EHR implementation has any influence on educational practices, is there anything else you think we should know that we have not talked about?
